# Distribution of Globe Excursions Within the Orbits Monitored by Eye Tracking Glasses in Ambulatory Subjects Engaged in Their Normal Daily Activities

**DOI:** 10.1167/iovs.66.3.20

**Published:** 2025-03-10

**Authors:** Yicen J. Zheng, Thomas N. Gentry, John R. Economides, Jonathan C. Horton

**Affiliations:** 1 Program in Neuroscience, Department of Ophthalmology, University of California, San Francisco, San Francisco, California, United States

**Keywords:** ductions, vergence, gaze angle, saccade, Tobii Pro Glasses 3

## Abstract

**Purpose:**

It is unknown how gaze angle deviates over the course of normal daily activities, and whether its distribution is affected by vergence. To address these issues, an eye tracker was used to record eye positions in ambulatory subjects engaged in their usual pursuits.

**Methods:**

Twenty-seven normal subjects with a mean age of 23.6 years (range, 4–68 years) wore the eye tracking glasses, generating 328 min/person of usable data. Histograms were compiled to show the distribution of (1) horizontal gaze angles, (2) vertical gaze angles, and (3) vergence.

**Results:**

The histogram of horizontal gaze angles showed a bimodal distribution of vergence, with a distance peak at 1.6° and a near peak at 7.6°. The mean standard deviation of eye position was greater during far viewing (8.93°) than near viewing (6.65°). Horizontal eye position deviated by more than 25° from primary position less than 1% of the time. Vertical eye position shifted from a mean of −3.58° during far viewing to −8.54° during near viewing. Overall, the standard deviation of vertical eye position (11.63°) was greater than that of horizonal eye position (8.41°).

**Conclusions:**

Deployment of a mobile eye tracker has revealed four main findings about eye position in the orbit. First, horizontal gaze angle remains within ±16.8° of primary gaze 95% of the time, reflecting an aversion to large horizontal ocular excursions. Second, mean vertical gaze position is shifted downward (−5.19°). Third, increased vergence is associated with a gaze shift downward. Fourth, increased vergence reduces the distribution of horizontal eye positions.

The extraocular eye muscles rotate the globes in their orbits. The foveae alight on targets of interest and maintain fixation when movement occurs of either the target or the head.^1^ The extreme limits of voluntary globe excursion were reported by Helmholtz and other investigators (reviewed by Duke-Elder and Wybar^2^). Their studies have been repeated in the modern era, with essentially the same results. The eyes can be moved from primary position approximately 50° horizontally and downward.^3^^–^^6^ Upgaze is slightly more limited, and becomes progressively more so with age.^7^^–^^11^ There is considerable variability in performance among normal subjects, depending on effort, orbital anatomy, axial length, and other factors.^12^

The term “gaze angle” refers to the direction of the line of sight, summing movements of the eye in the orbit and the head/body in space.^1^ Surprisingly, humans are loath to take advantage of the full range of eye movements within the orbit that are available to them.^13^ Instead, shifts in gaze usually combine eye, head, and body movements.^14^^–^^19^ The relative contribution of the head movement depends on the amplitude of the gaze shift, with some variability arising from individual propensities.^20^^,^^21^ Stahl^22^ has shown with eye movement recordings in the laboratory that if horizontal saccades from primary gaze are less than 20°, there is generally little head movement. Gaze shifts beyond ±20°, however, are accompanied by increasing head swivel. One hardly gives much thought to this behavior until cervical rotations are limited by a stiff neck. In that case, one must compensate by rotating the entire body to avoid making large eye movements, which are uncomfortable.

The invention of eye trackers that can be worn by mobile subjects has yielded insights into human ocular motor behavior in natural settings, rather than under artificial laboratory conditions.^23^^–^^29^ Foulsham et al.^30^ recorded horizontal and vertical eye positions in 14 subjects wearing an eye tracker while walking around a university campus. Horizontal gaze position was normally distributed around primary orbital position, with a standard deviation of 7.6°. Vertical gaze position was centered 10° above the horizon, with a standard deviation of only 5.3°. This innovative study confirmed that it is natural behavior to keep the eyes relatively close to primary position. Gaze angle deviated beyond 15° only about 5% of the time.

Only a single eye was tracked in the Foulsham study, so vergence behavior could not be explored. In a recent study, we recorded vergence angle with a wearable binocular eye tracker in a cohort of ambulatory subjects.^31^ The main finding was that people display a bimodal distribution of fixations—near or far—with less time spent fixating at intermediate distances. In the present study, we investigate the impact of vergence angle on the distribution of versions made by normal subjects engaged in their regular repertoire of daily activities.

## Methods

### Experimental Subjects

This study was approved by the University of California San Francisco Institutional Review Board and followed the principles of the Declaration of Helsinki. Adult subjects gave informed written consent to participate. Minors expressed verbal assent and a parent provided formal informed consent.

The 27 subjects who wore the eye tracking glasses included the 4 authors, their professional colleagues, family members, friends, and neighbors. Most individuals wore the glasses as a favor or because they were curious about our research. There was no organized recruitment strategy. All subjects had normal visual function. In some subjects, this was verified by an ophthalmological screening examination, including the assessment of acuity, pupils, eye movements, and stereopsis. For others, we relied on the subject's assurance that their vision was normal. No participant had strabismus, corneal disease, nystagmus, prior eye surgery, or cervical spine disease. Most subjects were recorded without refractive correction. Individuals with a large refractive error were corrected with spherical equivalent lenses that fit into the glasses frames or wore their contact lenses.

The 27 subjects were asked to wear the eye tracking glasses for as long as convenient while engaged in their regular, daily activities. Subjects removed the eye tracking glasses when using the bathroom. The device resumed tracking with no loss of calibration when placed back on the head.

### Eye Position Measurements

**Figure 1.**
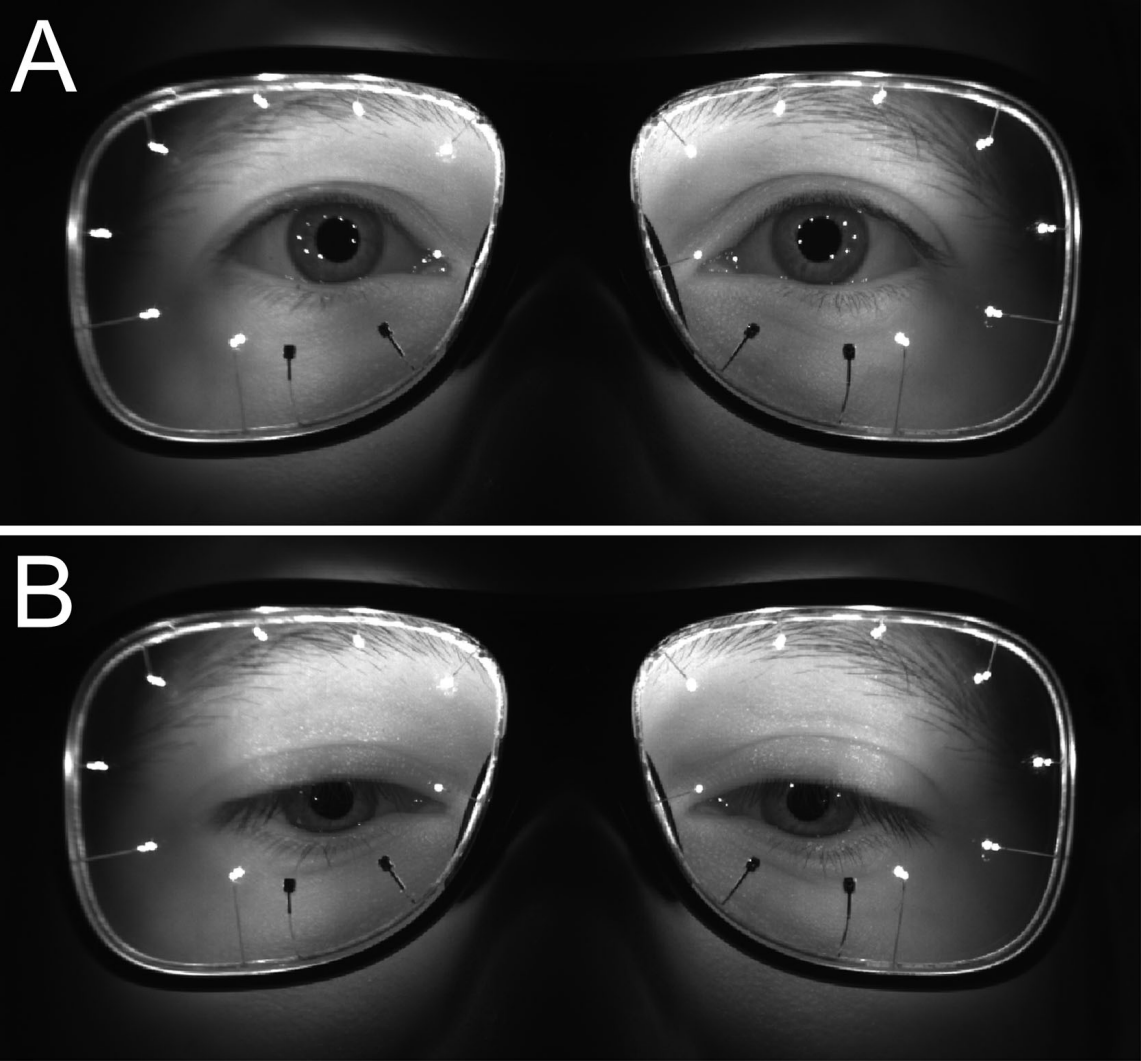
Tobii Pro Glasses 3. (**A**) Infrared image of a subject in primary gaze wearing the eye tracker. It contains eight miniature infrared illuminators and two cameras (inferonasal) in each lens. The eight illuminators are reflected on the cornea in a semicircular pattern. The scene camera and microphone are mounted on the bridge of the glasses. After calibration, the unit continues to record accurately even if one camera and up to four illuminators are occluded in each lens. (**B**) Subject in 25° downgaze, at the point where his tracking was lost. The upper lid is clipping the pupil and only three illuminator reflections are visible.

Measurements of eye position in ambulatory subjects were obtained with Tobii Pro Glasses 3 (www.tobiipro.com). It consists of a pair of eye glasses, containing eight infrared illuminators and two cameras embedded in each plano lens (Fig. 1A). The device has a scene camera that captures 95° horizontally by 63° vertically at 25 Hz. The glasses are connected via a cable to a recording unit. After calibration, the fixation point for each eye relative to the scene is computed at 50 Hz from locations of the pupil center and the eight illuminator reflections on the cornea. It is overlaid on the view provided by the scene camera and displayed on a monitor. Data are saved to an SD card in the recording unit for later analysis.

Testing in our laboratory has shown that the Tobii Pro Glasses 3 capture horizontal eye position within ±45° of primary gaze. Upgaze can be tracked up to 35° above primary gaze. In downgaze, the descent of the upper eyelid causes clipping of the pupil (depending on its diameter) and loss of most illuminator reflections (Fig. 1B). As a result, eye position recordings in downgaze are usually truncated at about −25°. There is also a small error in the vergence angle reported by the Tobii Pro Glasses 3. In a previous study, we encountered an average offset of +1° in the value of vergence angles measured between 0.25° and 8°.^31^

Constriction of the pupils induced by changes in light can be reported incorrectly by some eye tracker models as a change in vergence angle.^32^ To assuage concern that the Tobii Pro Glasses 3 might suffer from this defect, five subjects were recorded while fixating steadily on a target requiring 5° of convergence. Pupil diameter was modulated over a range of 2 mm (usually between 5 mm and 3 mm) by repeated on/off cycles of ambient lighting. Vergence angle recorded by the instrument showed no fluctuation that correlated with changes in pupil size (Supplementary Fig. S1). It appears that the Tobii Pro Glasses 3 are free of this potential source of artifact.

The lithium ion battery that powers the Tobii Pro Glasses 3 lasts only 1.5 hours. To obtain longer ambulatory recordings, the recording unit was connected to an external rechargeable battery pack. This arrangement permitted up to 12 hours of ambulatory recording. When the unit detected that the batteries were nearly exhausted, an automatic shutdown mode was triggered, saving the data. The recording unit and power pack were fitted into a small padded knapsack worn by the subject. Each device was only 1 inch thick, allowing subjects to sit comfortably in a chair or car seat with the knapsack on their back. For outdoor activities, subjects used slip-on sunglasses provided by the manufacturer to avoid washing out the illuminator reflections.

### Data Analysis

Using a custom script written in Igor Pro (www.wavemetrics.com), data were extracted from the JSON file encoding the three dimensional vector (*x, y, z*) representing the gaze direction of each eye. The horizontal position of the fixation point for each eye in degrees was calculated from these vectors by applying the following functions, given *x*, *z* ∈ (0, 1) ⊂ R:

$$\;\mathrm{Horizontal}\;position_{right\;eye}=-\arctan\left(\frac{x_{right\;eye}}{z_{right\;eye}}\right)\times \frac{180^{\circ }}{\pi }$$

$$\;\mathrm{Horizontal}\;position_{\mathrm{left}\;\mathrm{eye}}=-\arctan\left(\frac{x_{left\;eye}}{z_{left\;eye}}\right)\times \frac{180^{\circ }}{\pi },$$

where *x* is the horizontal component and *z* is the depth component of the gaze direction vector.

The vertical position of the fixation point for each eye in degrees was calculated from these vectors by applying the following functions, given *y*, *z* ∈ (0, 1) ⊂ R:

$$\;Vertical\;position_{right\;eye}=-\arctan\left(\frac{y_{right\;eye}}{z_{right\;eye}}\right)\times \frac{180^{\circ }}{\pi }$$

$$\;Vertical\;position_{\mathrm{left}\;\mathrm{eye}}=-\arctan\left(\frac{y_{left\;eye}}{z_{\mathrm{left}\;\mathrm{eye}}}\right)\times \frac{180^{\circ }}{\pi },$$

where *y* is the vertical component and *z* is the depth component of the gaze direction vector.

To calculate vergence, the horizontal position of the right eye was subtracted from the horizontal position of the left eye. Positive values denoted convergence. Eye position was calculated by taking the mean of the right and left eye positions. Negative values denoted left gaze or downgaze. Histograms were compiled to show the distribution of eye positions or vergence angles. As head movements were not measured in this study, “gaze angle” is used colloquially in this report as a synonym for position of the eye in the orbit.

The data stream from the eye tracking glasses contains interruptions and aberrant points, owing to transient loss of tracking in one or both eyes. We applied two remedial filters.^31^ The first filter interpolated over gaps lasting up to 25 samples, with the median value of the surrounding 24 samples. This eliminated blinks and other brief artifacts. The second filter removed spurious readings by comparing the value of each point with the 24 points surrounding it. If more than 1° outside the median, it was replaced with the median value.

The custom software used to analyze the Tobii data files is available at Open Science Framework via this link: https://osf.io/fesdp.

## Results

**Figure 2.**
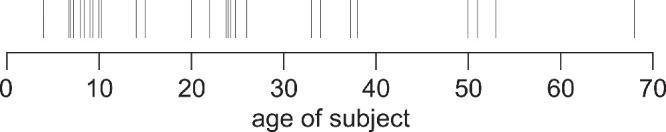
Distribution of ages of the 27 subjects in this study.

There were 14 males and 13 females in the study population, ranging between 4 and 68 years in age (Fig. 2). The mean age was 23.6 ± 17.1 years. The mean duration of recording was 347.4 ± 192.6 minutes (range, 68.6–677.4 minutes), after excising epochs when the tracker was removed for breaks. This duration yielded a mean of 258.5 minutes of unfiltered data and 327.8 minutes of filtered data. Application of the filters improved the data yield from 74% to 94%.

**Figure 3.**
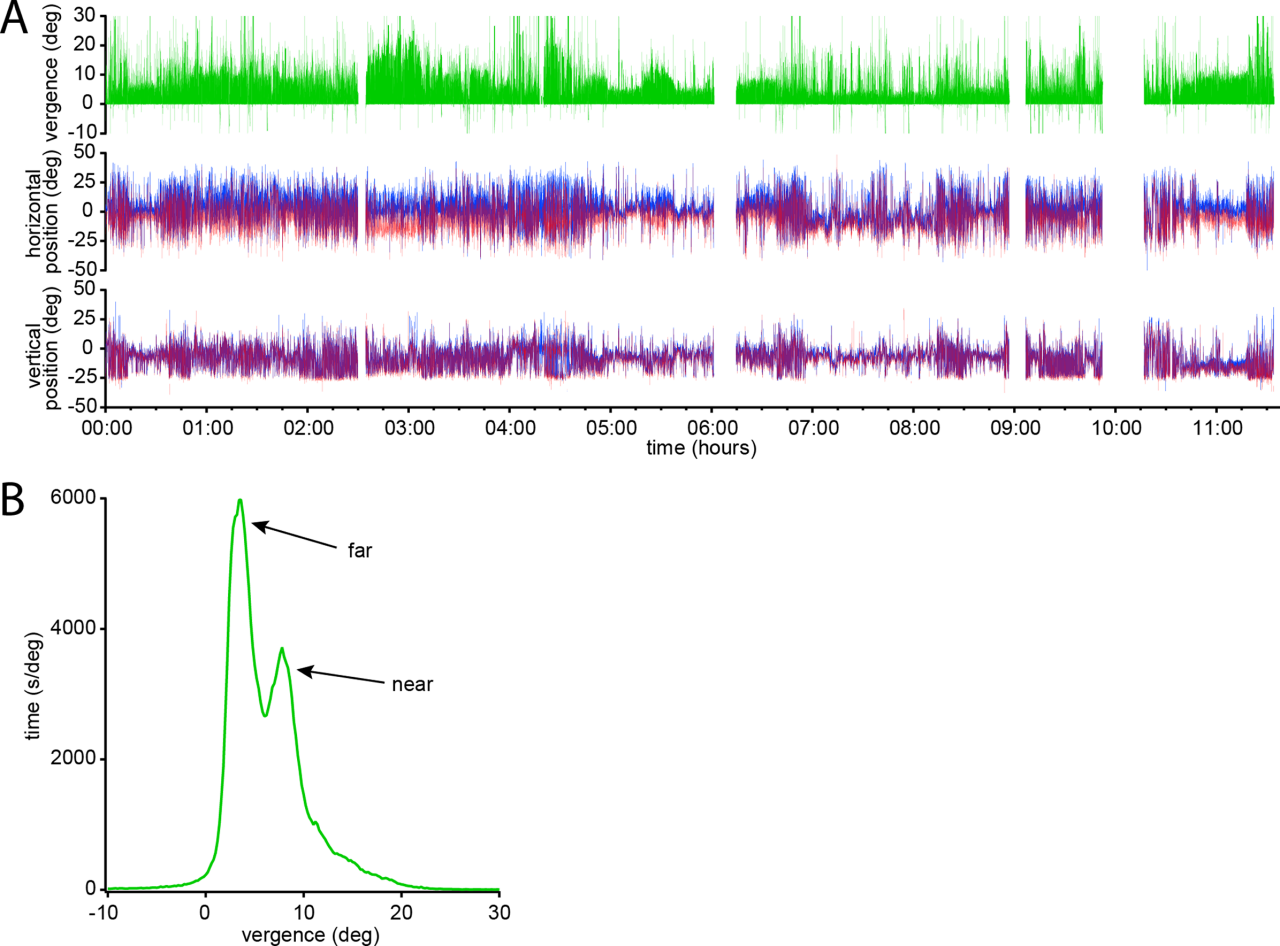
Example of data recorded over nearly 12 hours from a 24-year-old woman. (**A**) *Green trace* shows vergence angle (positive values denote convergence). Gaps in the record correspond with periods when the tracking glasses were removed. Traces below show the horizontal and vertical position of each eye (red = right eye; blue = left eye), with positive values indicating right gaze or upgaze. After removing gaps, there were 640.2 minutes of data. The unfiltered duration was 504.0 minutes and the filtered duration was 615.6 minutes. (**B**) Histogram of the vergence data in (**A**), showing bimodal distribution, with peaks at 3.6° (mode of distance fixations) and 7.8° (mode of near fixations). Due to tracker error, vergence values should be offset by −1°. The negative values forming the left sided tail, representing exotropia, are all spurious and amount to 11.5 minutes (2%).

Figure 3A shows data from a subject, chosen because she wore the tracker for 11.55 hours, a period that approached the maximum battery life of 12 hours. She took 4 breaks, lasting a total of 53 minutes, leaving 640.2 minutes of net recording. Her vergence angle, horizontal eye position, and vertical eye position varied systematically as her work day activities switched between cooking, eating, driving, walking, using a computer, holding a meeting, attending a seminar, using a smartphone, jogging, and so on.

Figure 3B shows the profile of vergence behavior captured by the data stream, which lasted 615.6 minutes after filtration. The distribution was bimodal, with the taller peak (3.6°) corresponding to distance viewing and the lesser peak (7.8°) to near viewing. A 1° correction was applied to these values to compensate for tracker error in vergence measurement (Methods), yielding a mean vergence of 2.6° for distance viewing and 6.8° for near viewing. The subject's interpupillary distance was 60 mm. Therefore, these vergence angles corresponded with viewing at mean distances of 132 cm and 50 cm.

**Figure 4.**
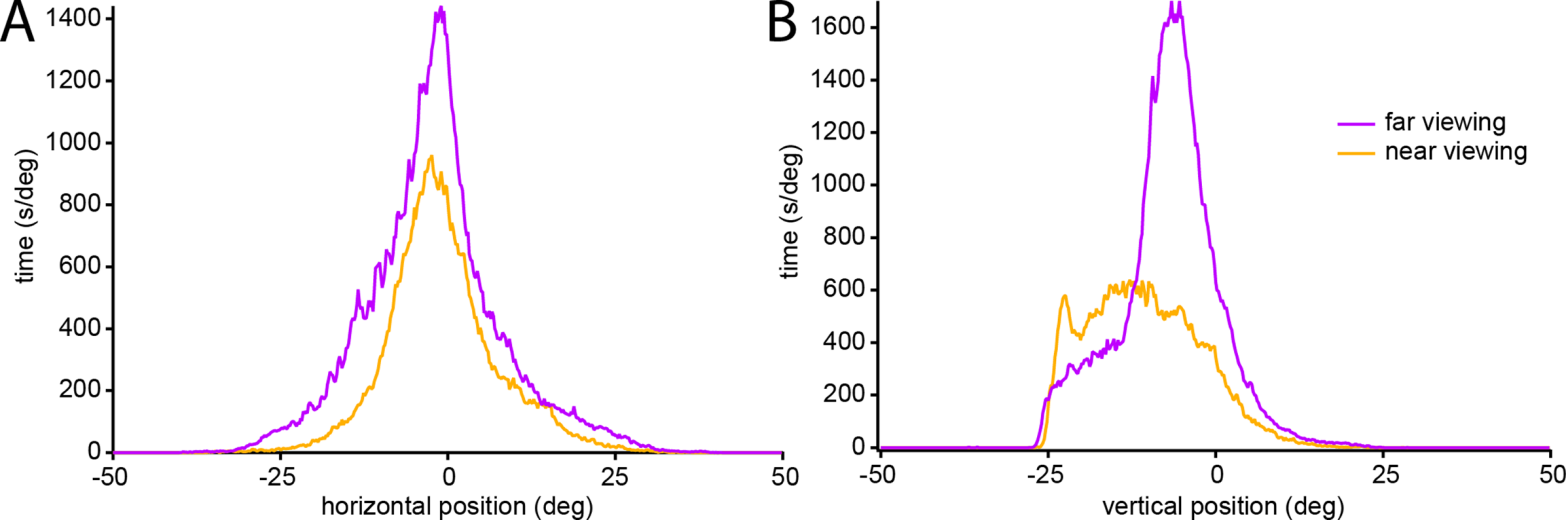
Histograms of gaze position. (**A**) Horizontal gaze position, dividing the data into far and near viewing, by splitting vergence angle at the notch in Figure 3B. During far viewing, the distribution of eye positions is relatively narrow (SD 9.98°), rarely extending beyond 25° from primary gaze. During near viewing the distribution is even more narrow (SD 8.33°). (**B**) Vertical gaze position, also divided into far and near viewing. The mean position is at −7.01 ± 7.47° for distance viewing and −10.63 ± 8.37° for near viewing. Sharp cutoff near −25° is an artifact from loss of eye tracking in extreme downgaze (see Methods).

A glance at the eye positions recorded in Figure 3A reveals that they seldom strayed more than 25° from the primary orbital position (0°). To quantify the distribution of eye positions, the vergence angle (Fig. 3B) was divided at the saddle notch into distance and near fixations. The horizontal eye position was then plotted for distance and near viewing (Fig. 4A). When viewing at far, the standard deviation of eye positions was 9.98° (*n* = 1,137,460 data points). When viewing at near, the standard deviation was 8.33° (*n* = 709,345 data points). Only 33,791 of 1,846805 horizontal eye positions (1.8%) were recorded more than 25° from primary gaze.

The same analysis was conducted for vertical eye position (Fig. 4B). When viewing at far, the mean vertical gaze position was −7.01 ± 7.47°, well below primary gaze (0°). When viewing at near, it shifted even lower, to a value of −10.63 ± 8.37°. The true downward bias, both at far viewing and near viewing, might have been under sampled because eye tracking was usually lost below −25°.

**Figure 5.**
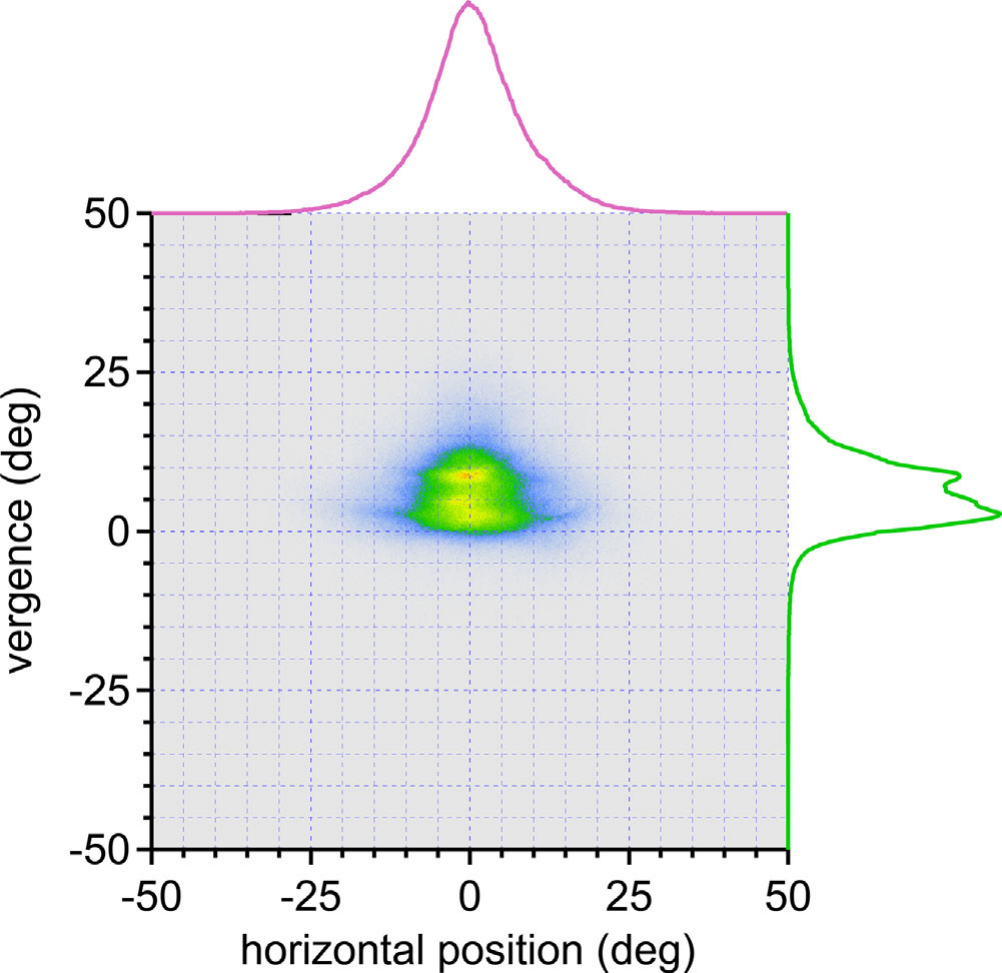
Bivariate histogram (0.1° square bins) showing horizontal eye position (*pink trace*, top) vs. vergence angle (*green trace*, right) for all 27 subjects. The histogram contains a trough at 7° (corrected value 6°) between the peaks of the vergence angle that occur for near and far viewing. The distribution of points becomes narrower with increased vergence.

Data from all 27 subjects confirmed a bimodal distribution of vergence angles, with a distance peak at 2.6°, a near peak at 8.6°, and a saddle at 7.0° (Fig. 5). To give subjects equal weight, despite differences in the length of their eye tracking session, a subsample of 180,000 data points (1 hour) was extracted randomly from each subject's filtered data for all population analyses. Incorporating a 1° offset for tracker error, the corrected values were 1.6°, 7.6°, and 6.0°. The mean interpupillary distance of the 27 subjects was 60.3 ± 4.6 mm, corresponding with far viewing at a mean distance of 216 cm and near viewing at a mean distance of 45 cm.

**Figure 6.**
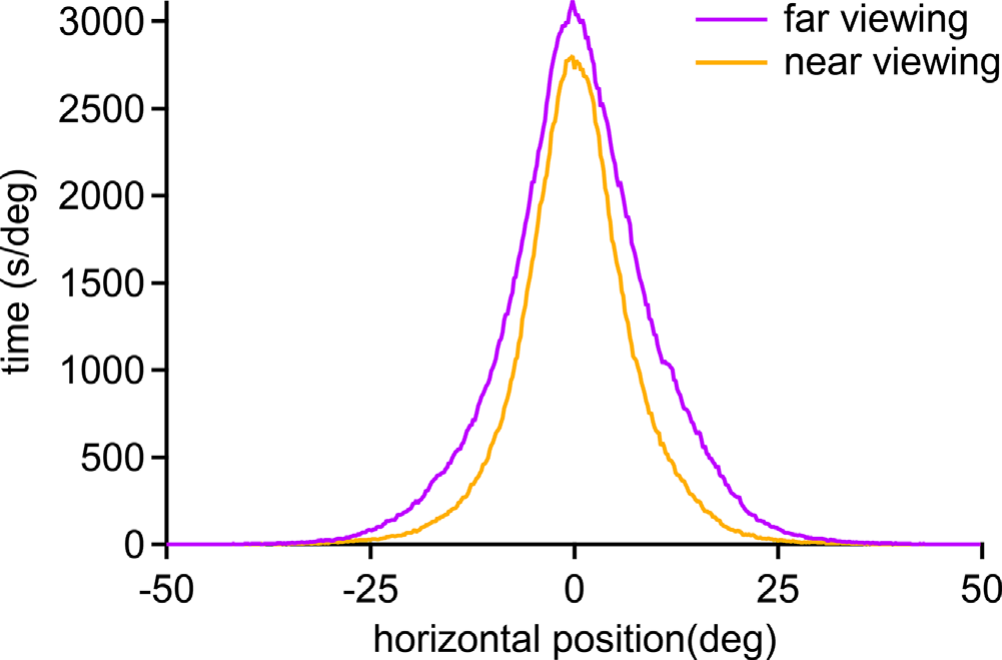
Data in Figure 5, replotted to show horizontal eye position for all subjects during far vs. near viewing. The standard deviation was calculated for each subject's eye position during far and near viewing. The mean value of these standard deviations during far viewing (8.93°) was greater than during near viewing (6.65°). The range of potential eye positions was ±50°, but less than 1% of eye positions were recorded more than 25° from primary gaze (0°).

Horizontal eye position for all subjects was broken at the notch value of 6° vergence into far (59% of the time) viewing and near (41% of the time) viewing (Fig. 6). When viewing at far, the mean eye position was 0.96 ± 2.66° (Table). When viewing at near, the mean eye position was 0.29 ± 3.14°. The mean eye positions were not significantly different at far vs. near (*P* = 0.25, paired *t* test; *n* = 27). For each subject, the standard deviation was calculated for the distribution of eye positions. For far eye positions, the mean standard deviation was 8.93 ± 1.39°. For near eye positions, the mean standard deviation was 6.65 ± 1.41°. The mean standard deviation was significantly greater for far eye positions compared with near eye positions (*P* < 0.0001, paired *t* test; *n* = 27).

**Table.** Mean Values for Horizontal and Vertical Eye Positions for the 27 Subjects, Divided Into Near Viewing vs. Far Viewing at a Vergence Angle of 6°

	Horizontal Eye Positions						Vertical Eye Positions					
	All Data		Near Viewing		Far Viewing		All Data		Near Viewing		Far Viewing	
Subject	Mean	SD	Mean	SD	Mean	SD	Mean	SD	Mean	SD	Mean	SD
1	0.37	7.38	0.50	6.30	0.26	8.21	−14.43	8.70	−15.73	7.21	−13.27	9.68
2	−0.33	4.98	0.50	4.34	−1.70	5.62	−9.70	6.80	−10.65	5.86	−8.11	7.88
3	−0.43	8.73	−0.39	7.62	−0.54	11.13	−16.95	11.24	−19.98	9.84	−9.02	10.79
4	−2.71	6.05	−2.60	5.46	−2.81	6.55	2.52	11.31	3.84	10.81	1.32	11.62
5	2.38	8.95	6.27	8.52	2.22	8.93	7.89	10.29	−3.60	16.32	8.38	9.66
6	−0.24	6.50	−1.10	5.28	−0.16	6.60	7.69	7.41	−3.96	8.35	8.79	6.28
7	0.21	7.31	−0.33	5.60	1.39	9.96	−5.26	8.93	−5.74	8.52	−4.25	9.67
8	3.40	6.84	2.92	4.58	3.75	8.07	−8.71	8.35	−12.88	6.01	−5.69	8.51
9	1.77	9.05	3.85	6.96	0.72	9.77	−0.85	10.77	−7.22	10.30	2.35	9.50
10	1.24	6.19	0.35	4.42	2.75	8.15	−7.71	7.50	−8.57	7.43	−6.24	7.39
11	−1.64	7.11	−2.26	5.45	−1.45	7.53	−11.93	7.58	−14.00	7.47	−11.31	7.51
12	−0.75	8.53	0.23	6.86	−0.91	8.75	3.98	7.99	−2.36	8.03	4.95	7.52
13	0.77	9.26	0.06	7.68	1.14	9.96	−23.22	9.30	−27.10	9.23	−21.19	8.67
14	−1.49	9.42	−0.74	8.32	−1.96	10.01	−8.41	8.03	−10.65	8.39	−7.01	7.47
15	4.75	7.99	4.41	7.64	10.85	11.09	3.07	9.80	2.85	9.69	7.06	10.91
16	−0.07	9.39	−1.08	9.16	0.69	9.52	−4.48	10.44	−2.42	10.12	−6.05	10.41
17	5.61	7.79	7.00	6.79	4.05	8.50	−9.75	9.10	−11.43	8.76	−7.87	9.10
18	0.01	7.69	1.11	7.21	−0.73	7.92	−9.18	8.39	−9.31	9.24	−9.10	7.77
19	2.80	8.79	2.47	5.68	2.89	9.46	0.29	8.37	−4.61	7.06	1.60	8.20
20	−3.43	8.83	−4.69	7.10	−2.38	9.94	−9.12	11.04	−13.45	10.22	−5.48	10.36
21	−1.67	8.48	−4.13	6.84	1.90	9.31	2.56	11.29	1.07	11.82	4.73	10.09
22	0.06	9.40	−0.88	7.15	0.35	9.97	−6.36	10.29	−15.83	9.27	−3.46	8.72
23	0.92	8.88	0.34	5.24	1.24	10.37	−2.19	10.08	−3.94	9.09	−1.20	10.48
24	1.53	8.62	4.24	7.06	1.02	8.79	−4.95	8.57	−10.60	9.13	−3.87	8.02
25	0.15	8.36	−0.09	8.22	0.38	8.47	−10.86	7.14	−10.90	7.83	−10.82	6.42
26	−0.51	6.17	−1.34	4.95	1.12	7.76	−1.20	5.28	−1.61	5.10	−0.42	5.53
27	0.73	10.87	−6.67	9.10	1.82	10.68	−2.83	8.28	−11.69	9.63	−1.54	7.21
means	0.50	8.06	0.29	6.65	0.96	8.93	−5.19	8.97	−8.54	8.92	−3.58	8.72
SD	2.05	1.33	3.14	1.41	2.66	1.39	7.39	1.56	7.02	2.15	7.09	1.57

**Figure 7.**
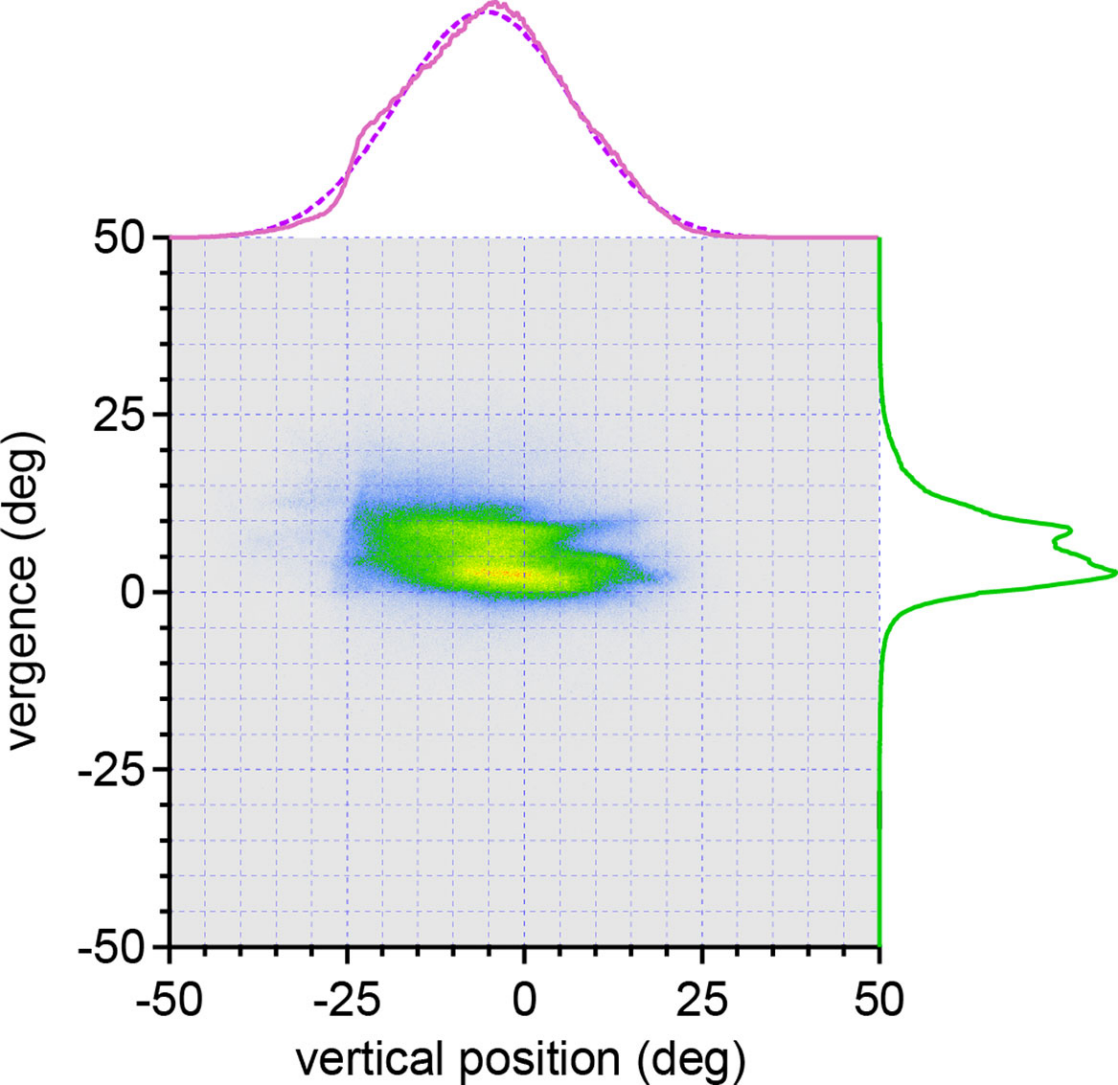
Bivariate histogram showing vertical eye position (*pink trace*, *top*) vs. vergence angle (*green trace*, *right*) for the population. The distribution of points is wider than for horizontal gaze and their mean value (−5.19°) is displaced downward (leftward on the *x* axis). A Gaussian fit (*dashed line*) to the distribution has a peak at −5.24°, indicating that the truncation of recordings at −25° in some subjects because of hooding of the cornea by the upper eyelid skewed only slightly the mean vertical position.

Figure 7 shows the relationship between vertical eye position and vergence angle for the entire population. When viewing at far, the mean eye position was −3.58 ± 7.09° (Fig. 7). When viewing at near, the mean eye position was −8.54 ± 7.02° (Table). The mean vertical eye position was significantly higher at far vs. near (*P* < 0.0001, paired *t* test; *n* = 27), indicating that an increase in vergence is accompanied by a downward shift in mean vertical eye position. For each subject, the standard deviation was calculated for the distribution of eye positions. For far eye positions, the mean standard deviation was 8.72 ± 1.57°. For near eye positions, the mean standard deviation was 8.92 ± 2.15°. The mean standard deviation for far eye positions was statistically indistinguishable from the mean standard deviation for near eye positions (*P* = 0.58, paired *t* test; *n* = 27).

A plot of the horizontal eye position vs. the vertical eye position for the 27 subjects shows a vertical ovoid cloud (Fig. 9). The distribution of left/right eye positions is distributed symmetrically around 0.50°. The 4,860,000 horizontal eye positions have a standard deviation of 8.41°, meaning that less than 5% of the time they stray more than 16.82° from primary position. Only 47,152 (0.97%) are located more than 25° from primary position. Vertical gaze is displaced downward, with a mean position of −5.19°. The 4,860,000 vertical eye positions have a standard deviation of 11.63°.

Inspection of individual traces shows that the mean horizontal position varies less than the mean vertical position (Fig. 10). The mean of all subjects’ horizontal positions was 0.50°. It was not located exactly at 0°, perhaps because individual subjects engaged in activities biased slightly to either side. It is also possible that the tracker contributed a small offset. The mean absolute difference between each subject's mean horizonal position and the population mean was 1.51°. Vertical eye positions showed greater variability, with the mean absolute difference between each subject's mean and the population mean equal to 5.85°.

## Discussion

The invention of eye trackers has spawned a huge body of research addressing the ocular motor behavior of subjects viewing various displays, photographs, or movies of natural scenes.^33^ The main thrust has been to define image features that attract fixations to optimize efficient extraction of information. Most work has been done in a laboratory setting with stationary equipment, but more recently, the development of eye trackers that can be worn by ambulatory subjects has made it possible to examine ocular motor behavior while subjects carry out everyday tasks. Recordings have been made of people walking, crossing a street, making a cup of tea or a sandwich, caring for patients, cooking, and driving.^27^^,^^34^^–^^42^ As in the laboratory, the main goal has been to understand how eye movements facilitate the execution of different motor and perceptual tasks.^43^

Although many ambulatory recordings have been reported, relatively little attention has been paid to the distribution of eye positions during natural visual behavior. Research efforts have focused on documenting where the eyes are directed in the visual scene, rather than where they are located in the orbits. Addressing the latter point, however, is important for understanding how eye, head, and trunk movements are coordinated. It is also important for understanding the impact of diseases, such as Graves’ orbitopathy, that limit eye movements. Four principal findings have emerged from these recordings of orbital eye position made in subjects while engaged in their normal gamut of activity.

The first finding is that horizontal eye positions are relatively limited, when one considers that they have the potential to range up to ±50° from primary orbital position. Our recordings yielded an approximately normal distribution around primary gaze, with a standard deviation of 8.41° (Fig. 9). The eyes were rotated beyond 25° less than 1% of the time. These findings agree closely with Foulsham et al.,^30^ who reported that horizontal eye position had a standard deviation of 7.6°. The reason for such a strong center bias is uncertain. Large horizontal eye rotations cause an unpleasant tugging sensation and even exert traction on the optic disc.^44^ Eccentric gaze requires tonic contraction of the rectus muscles, to overcome the elastic forces pulling the globe back into primary position. Maintenance of a deviated posture induces fatigue, manifested by end point nystagmus. The later phenomenon often occurs at angles considerably less than maximal and destabilizes gaze.^45^ Finally, large ocular excursions may be infrequent simply because the gaze control system usually couples eye and head movements through a common motor command.^14^^,^^16^^–^^18^

The second finding is that vertical eye position, unlike horizontal eye position, is not symmetrically distributed around primary orbital position. There was a mean displacement of −5° (Fig. 9). Foulsham et al.^30^ reported a mean vertical position of +10°. This discrepancy can be explained by the fact that their cohort was smaller, sampled for less time, and all engaged in the same activity, namely, walking around a college campus. Vertical eye position is more variable than horizontal eye position (compare Figs. 10A and 10B), presumably because it is more task dependent. Walking around campus may favor upgaze, but viewing a smartphone, computer, reading, eating, or engaging in activities that require eye–hand coordination are more likely to occur in downgaze. The collective demands of the tasks carried out by our cohort resulted in a mean downward gaze shift of 5° and a greater standard deviation of vertical than horizontal eye positions (Fig. 9). It is curious that subjects do not negate the mean downward eye position of 5° by flexing the neck to keep the mean vertical eye position at primary position in the orbit. It is also noteworthy that vertical eye positions have a wider distribution than horizontal eye positions. This finding implies that individuals are more apt to make yaw, rather than pitch, adjustments of head or trunk posture to keep the eyes close to primary position.

**Figure 8.**
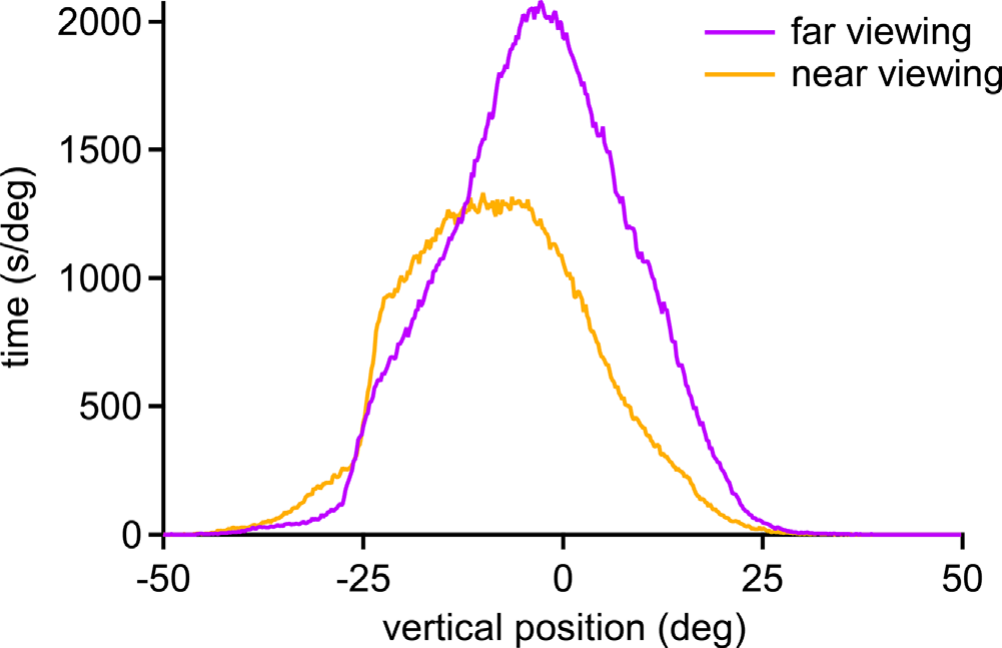
Data in Figure 7, replotting vertical eye position for entire population during far vs. near viewing. Mean vertical position shifts downward from −3.58° at far vergence to −8.54° at near vergence. The mean value of each subjects’ standard deviation of vertical eye position was nearly equal during far viewing (8.72°) and near viewing (8.92°). Sharp left shoulder to the curves is due in part to loss of eye tracking in downgaze below −25° in some subjects.

The third finding is that an increase in vergence angle is associated with a downward shift in the mean gaze position (Fig. 8^9^,^10^). This is such a common, intuitive experience that it scarcely requires comment. The hands have evolved as prehensile digital instruments under visual control. Given that they are located below the head, convergence on objects held in the hands usually requires a downward shift in gaze. This propensity explains why, in bifocal glasses, the segment for near viewing is located at the bottom of the lenses. Interestingly, the standard deviation of vertical eye position is not affected by the downward shift in the mean position that accompanies convergence (Fig. 8). This result indicates that both far and near activities generate similar variability in vertical eye positions.

**Figure 9.**
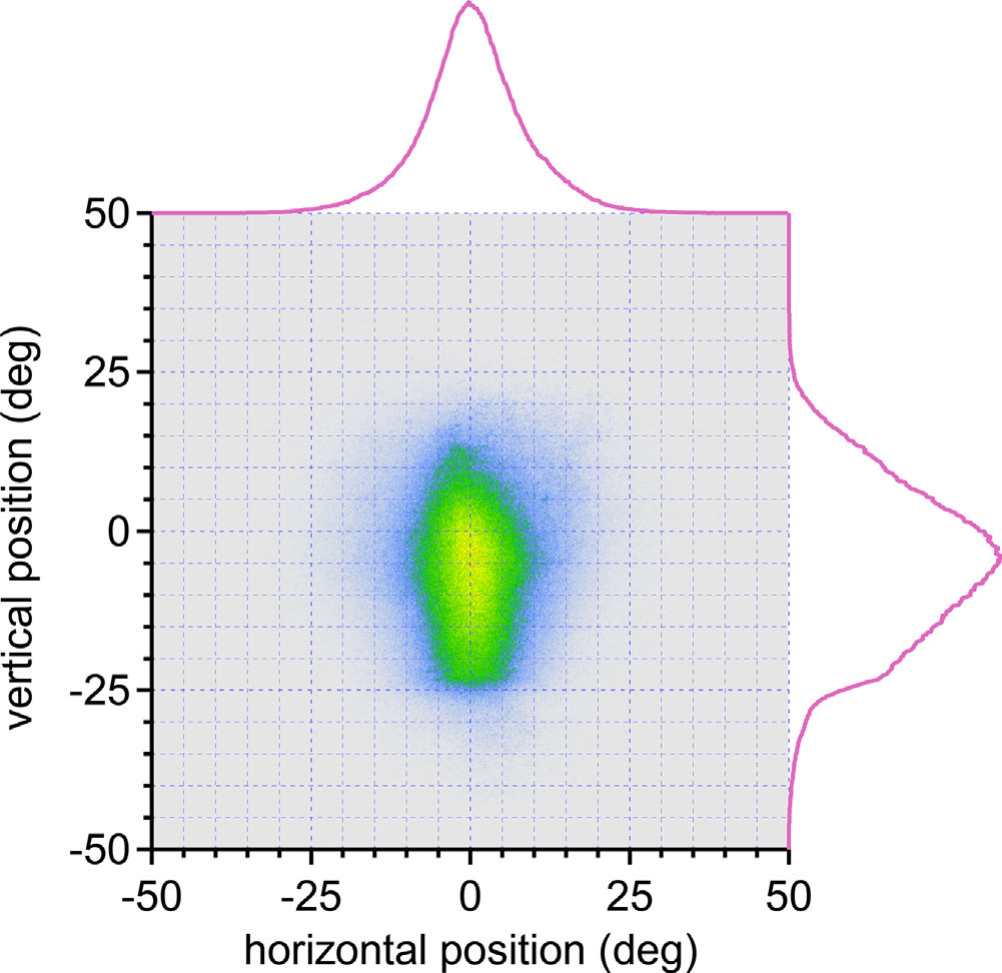
Bivariate histogram of horizontal eye position vs. vertical eye position for all 27 subjects. *Pink traces* combine data for far and near vergence in Figures 6 and 8. The distribution of eye position is much broader in the vertical (SD 11.63°) compared to the horizonal (SD 8.41°) axis. It is symmetrical around 0.50° with respect to left vs. right gaze, but displaced downward to a mean position of −5.19°.

**Figure 10.**
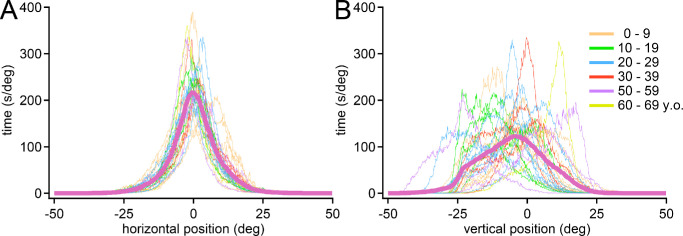
Individual histograms for the 27 subjects showing their (**A**) horizontal and (**B**) vertical eye positions. Among subjects, there is more heterogeneity in the distribution of vertical, than horizontal, eye positions. Subjects’ mean absolute difference from the population mean is 1.51° for horizontal eye position and 5.85° for vertical eye position. Purple line denotes mean of 27 individual traces.

The fourth finding is that an increase in vergence angle leads to a reduction in the variability of horizontal eye position. The mean of the subjects’ standard deviation of horizontal eye position was 8.93° at far viewing compared to 6.65° at near viewing (Fig. 6). For any prolonged near work, it is natural to orient the body and head to bring the task to the midline. Convergence on a near target, especially an object held in the hands, will maintain the eyes closer to primary horizontal position than when the eyes are diverged for distance viewing.

Shifts in vergence angle are usually accompanied, and indeed facilitated, by saccadic eye movements.^46^^–^^48^ Conjugate horizontal eye movements are generated by a signal sent to the abducens nucleus. Two populations of cells are activated: lateral rectus motoneurons and interneurons, which project contralaterally to medial rectus motoneurons via the medial longitudinal fasciculus. The act of converging results in the inhibition of the majority of both cell populations.^49^ As a result, it is more difficult to make lateral eye movements at moments when the eyes are also highly converged. This could explain, in part, why the standard deviation of eye positions is reduced at near viewing. Of course, as for any human behavior, it is difficult to determine whether action is driven by the demands of the task or represents a manifestation of capabilities inherent to the motor system. Both factors clearly shape human oculomotor performance.

The fact that humans seldom move their eyes beyond 25° from primary position helps to explain why mild restriction or paresis of an extraocular eye muscle can sometimes be asymptomatic. The eyes infrequently move far into the field of action of any given muscle. Consequently, patients are sometimes able to compensate for deficits discovered when full excursions are tested during diagnostic examination by simply making a head movement or adopting a head turn.

## Supplementary Material

Supplement 1
