# Supplementary material for: Distribution of Globe Excursions Within the Orbits Monitored by Eye Tracking Glasses in Ambulatory Subjects Engaged in Their Normal Daily Activities
Source: Invest Ophthalmol Vis Sci. 2025 Mar 10;66(3):20. doi: 10.1167/iovs.66.3.20 (PMC11905580; doi:10.1167/iovs.66.3.20)
Supplement: Supplement 1 [file iovs-66-3-20_s001.pdf]

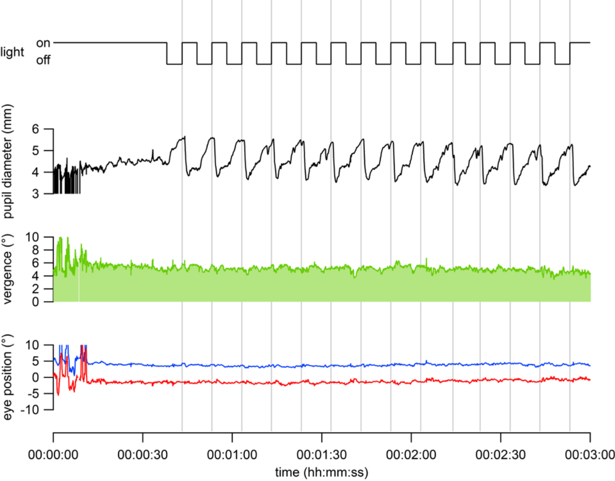

light
on
off
pupil diameter (mm)
6
5
4
3
vergence (°)
10
8
6
4
2
0
eye position (°)
10
5
0
-5
-10
00:00:00
00:00:30
00:01:00
00:01:30
00:02:00
00:02:30
00:03:00
time (hh:mm:ss)

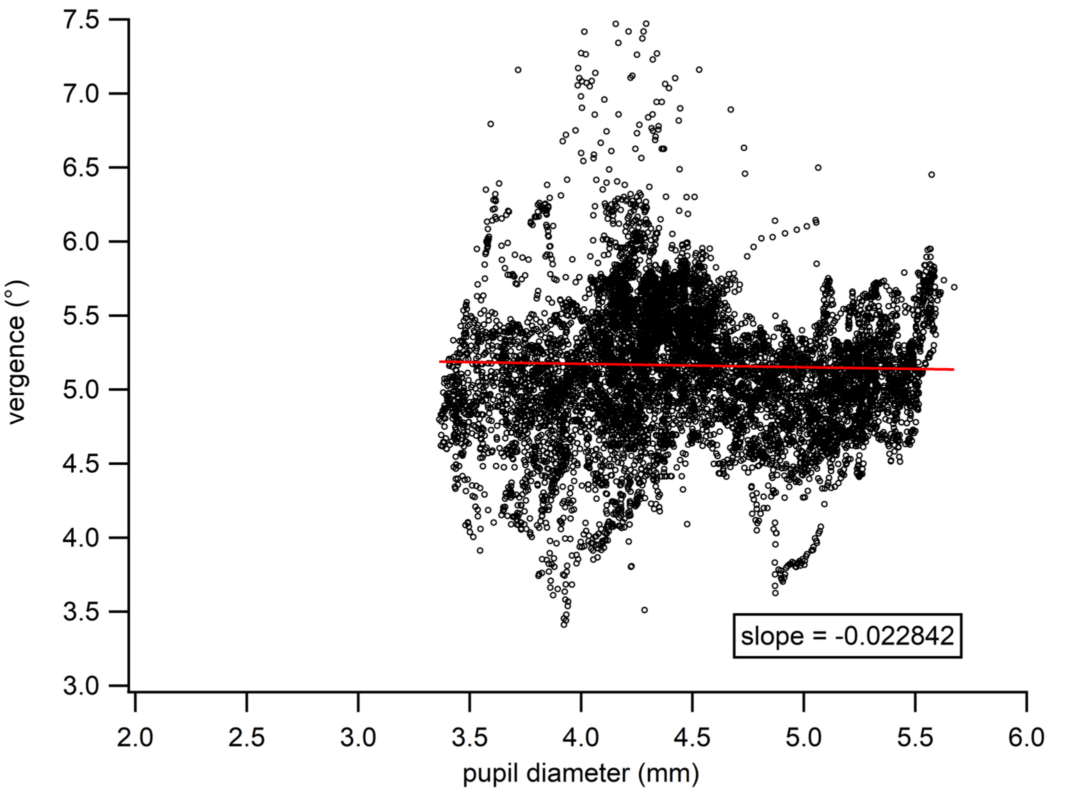

7.5
7.0
6.5
6.0
5.5
5.0
4.5
4.0
3.5
3.0
vergence (°)
2.0
2.5
3.0
3.5
4.0
4.5
5.0
5.5
6.0
pupil diameter (mm)
slope = -0.022842

Supplemental Figure 1. Changes in pupil diameter do not affect measurement of vergence angle. **A)** A 30-year-old subject fixated on a target requiring 5° of convergence while Tobii Pro Glasses 3 were used to recording pupil diameter, vergence angle, and the position of each eye (red = right eye, blue = left eye). The pupil size oscillated between 5 mm and 3 mm. There was some noise in the vergence measurement, but no fluctuation synchronized with changes in pupil diameter. **B)** Plot of vergence versus pupil diameter compiled every 20 msec from the data in (**A**). The slope is flat, excluding an impact of pupil diameter on vergence angle measurement.
